# Risk Reduction for End-Stage Renal Disease by Dietary Guidance Using the Gustatory Threshold Test for Salty Taste

**DOI:** 10.3390/nu12092703

**Published:** 2020-09-04

**Authors:** Yuki Ota, Mineaki Kitamura, Kiyokazu Tsuji, Kenta Torigoe, Ayuko Yamashita, Shinichi Abe, Kumiko Muta, Tadashi Uramatsu, Yoko Obata, Junya Furutani, Miwa Takashima, Hiroshi Mukae, Tomoya Nishino

**Affiliations:** 1 Department of Nephrology, Nagasaki University Hospital, 1-7-1 Sakamoto, Nagasaki 852-8501, Japan; yukiota@nagasaki-u.ac.jp (Y.O.); tsuji-kiyo@nagasaki-u.ac.jp (K.T.); ktorigoe@nagasaki-u.ac.jp (K.T.); ayamashita@nagasaki-u.ac.jp (A.Y.); k-io@nagasaki-u.ac.jp (K.M.); t-uramatsu@umin.ac.jp (T.U.); yobata-ngs@umin.ac.jp (Y.O.); tnishino@nagasaki-u.ac.jp (T.N.); 2 Department of Nephrology, Sasebo City General Hospital, 9-3 Hirase-cho, Sasebo, Nagasaki 857-8511, Japan; shinichiabe4227@gmail.com; 3 Department of Nutritional Management, Nagasaki University Hospital, 1-7-1 Sakamoto, Nagasaki 852-8501, Japan; furutani@nagasaki-u.ac.jp (J.F.); takasima@nagasaki-u.ac.jp (M.T.); 4 Department of Respiratory Medicine, Nagasaki University Graduate School of Biomedical Sciences, 1-7-1 Sakamoto, Nagasaki 852-8501, Japan; hmukae@nagasaki-u.ac.jp

**Keywords:** chronic kidney disease, educational hospitalization, gustatory threshold test, salty taste

## Abstract

Educational hospitalization of patients with chronic kidney disease (CKD) may slow the progression of renal dysfunction. However, the educational aspect that is more effective has not been identified to date. In this study, patients with CKD were evaluated for gustatory threshold for salty taste and received augmented salt reduction guidance under educational hospitalization at Nagasaki University Hospital from October 2016. In total, 277 eligible patients were enrolled and hospitalized from 2012 to 2019 (mean age of 69.2 years; men comprised 62.1%). We compared 141 patients (Group A) who were educated in the hospital after October 2016 and 136 patients (Group B) who received standard education in the hospital before October 2016. The changes in the estimated glomerular filtration rate (ΔeGFR) after hospitalization and dialysis induction rate within one year after hospitalization were evaluated. The ΔeGFR was significantly improved in Group A compared to Group B (A: 1.05 mL/min/1.73 m^2^/month, B: 0.55 mL/min/1.73 m^2^/month; *p* = 0.02). The dialysis induction rate was significantly lower in Group A than in Group B (A: 8.5%, B: 15.5%; *p* = 0.001). These trends were also observed by multivariate analyses. In conclusion, educational hospitalization with enhanced salt reduction guidance may reduce the risk of end-stage renal disease.

## 1. Introduction

Chronic kidney disease (CKD) results in the deterioration of renal function and can lead to end-stage renal disease (ESRD). CKD has a significant impact on patients’ prognosis and quality of life. In recent years, the prevalence of CKD has increased, with global estimates of 700 million patients with CKD and 1.2 million deaths due to CKD in 2017 [1]. Accordingly, there is an urgent need to address this growing public health concern. Several measures have been proposed to improve the prognosis of patients with CKD, including modification of diet and lifestyle. High salt intake is among the top dietary risk factors. Salt reduction is also beneficial for hypertension in cardiovascular diseases (CVD) [2]. Salt restriction is one such measure that has been reported to significantly decrease blood pressure in CKD patients [3]. A previous meta-analysis reported that salt restriction in patients with CKD decreased the amount of proteinuria [4]. Behavioral changes in patient lifestyles are needed to achieve salt restriction, and patient education is mandatory to support this. Generally, patients with CKD and a high risk for ESRD have fewer opportunities to receive suitable diet guidance due to several barriers, such as inadequate training, limited time of health providers, and the reluctance of patients [5]. Inpatient educational programs for patients with CKD may alleviate the progression of renal disease [6]. However, the type of educational program that is effective remains unclear. Incidentally, patients with CKD tend to have taste disturbance due to uremia, deficiency of zinc, peripheral nervous system disorder, and polypharmacy [7]. We hypothesized that educational hospitalization with enhanced salt reduction guidance would reduce the risk of ESRD. The aim of this study was, therefore, to elucidate whether our educational program, which included the evaluation of gustatory threshold for salty taste, significantly reduced the risk of ESRD.

## 2. Materials and Methods

### 2.1. Subjects and Study Design

Patients aged ≥20 years who were admitted to our hospital for the purpose of education on CKD between 2012 and 2019 were included in this study. Patients who could not be followed up with 1 year after the admission or died within 3 months after admission, cases with deteriorating general condition due to complications after admission, and patients with severe dementia were excluded. The definition of CKD was an estimated glomerular filtration rate (eGFR) less than 60 mL/min/1.73 m^2^ [8]. The new program, which included the evaluation of the gustatory threshold for salty taste in our facility, commenced in October 2016, and the gustatory threshold was not evaluated before October 2016. Patients admitted to our hospital after October 2016 were allocated to Group A, whereas those admitted to our hospital before October 2016 were allocated to Group B (as historical controls). Regarding Group A, the gustatory threshold for salty taste, blood pressure, and daily salt intake were evaluated upon admission and at 3 months after admission.

The data of Groups A and B were compared to elucidate whether intensive instructions regarding salt restriction based on the gustatory threshold for salty taste had a significant effect on the deterioration of renal function. Dialysis induction rates within 1 year and differences in the ΔeGFR at 6 months before and after admission were evaluated. The ΔeGFR was calculated based on the difference between eGFR at 6 months after admission and eGFR on admission, and divided by 6 to obtain the monthly rate. If dialysis was initiated in patients at 3 to 6 months after admission, eGFR just before the initiation of dialysis was used as a substitute for eGFR at 6 months after admission and divided by the number of months. The period for educational hospitalization was 1 week, and the educational program consisted of an evaluation of the risk factors for CKD progression, drug adjustments, diet and exercise guidance, and an introduction of renal replacement therapies. Education was carried out by multiple health professionals, such as doctors, nurses, pharmacists, dietitians, and physiotherapists. Dietitians provided a dietary instruction for ≥30 min, and the gustatory threshold for salty taste was examined at that time.

### 2.2. Assessments

#### 2.2.1. Estimated Glomerular Filtration Rate: eGFR

To calculate eGFR from serum creatinine, age, and sex, the following estimation equation for Japanese patients with CKD was used [9]: for men, eGFR (mL/min/1.73 m^2^) = 194 × age^−0.287^ × serum creatinine^−1.094^; for women, eGFR (mL/min/1.73 m^2^) = 194 × age^−0.287^ × serum creatinine^−1.094^ × 0.739 [9].

#### 2.2.2. Gustatory Threshold for Salty Taste

We investigated the gustatory threshold for salty taste in patients with CKD by using a sodium-impregnated test strip (SALSAVE; Advantec Toyo Co. Ltd., Tokyo, Japan), which was prepared with various salt concentrations (0.6, 0.8, 1.0, 1.2, 1.4, and 1.6 mg/cm^2^; normal range, ≤0.6 mg/cm^2^). A modified method was used to perform the evaluation [10,11]. First, the patients’ mouths were moistened with water and a strip with 0 mg/cm^2^ salt concentration was placed in their mouths for 3 s. Next, they were examined for salty taste, starting from the lowest concentration. Finally, the lowest recognized salt concentration was recorded as the measured value. If the patients were unable to recognize 1.6 mg/cm^2^ of salt, the gustatory threshold for salty taste was determined as 1.6 mg/cm^2^.

Salt intake was estimated using Tanaka’s method [12] based on the following formula:

24 h Na excretion (mEq/day) = 21:98 × {urinary sodium (mEq/L)/(urinary Cr   (mg/dL) × 10) × (−2.04 × age + 14.89 × body weight (kg) + 16.14 × height (cm) −  2244.45)}^0.392^                           

This method has been reported to be valid in patients with CKD and those using diuretics [13,14].

### 2.3. Statistical Analyses

Categorical variables are presented as number (%). Continuous variables are expressed as mean ± standard deviations. Non-normally distributed data are presented as median values with interquartile ranges. JMP 15 software (SAS Institute Inc., Cary, NC, USA) was used to perform statistical analyses. An unpaired or paired *t*-test and Mann–Whitney U-test (limited for nonparametric analyses) were used to compare continuous variables, and the chi-square test was used for categorical variables. Propensity score matching with a caliper coefficient set at 0.2 was performed to match Group A and B. To calculate the propensity score, age, sex, body mass index (BMI), diabetes history, eGFR, hemoglobin, systolic blood pressure, and degree of proteinuria were used as parameters because of their relevance to CKD treatment and data availability. Univariate and multivariate logistic regression analyses were also performed for both groups.

This study was approved by the ethics committee of the Nagasaki University Hospital (Nagasaki, Japan) (16020808-6) and performed according to the 1964 Declaration of Helsinki and its later amendments. Written informed consent was obtained from all patients in Group A. Patients in Group B were informed of the experimental procedures, but consent was not obtained given that their data were used as a historical control and collected from medical records. Included patients were anonymized. The ethics committee approved the waiver for consent.

## 3. Results

**Figure 1:**
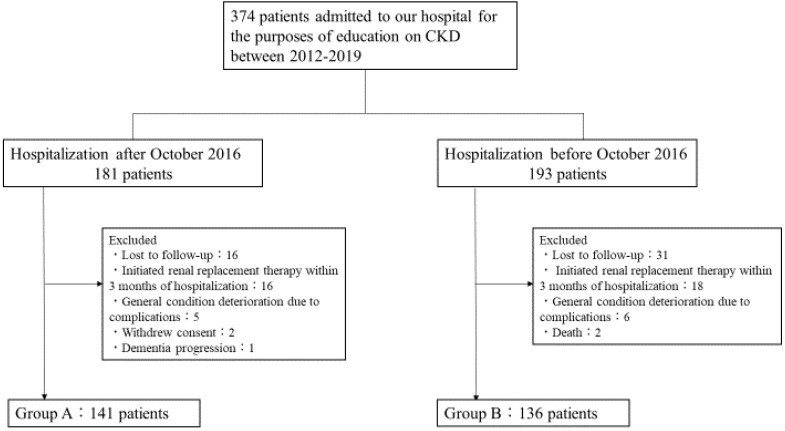
Flow chart of patients in the study groups.

In total, 374 patients were admitted to our hospital from 2012 to 2019 for the purpose of alleviating CKD progression. Renal replacement therapy was initiated in 34 patients within three months after admission. Two patients died, 11 patients’ general condition deteriorated due to complications during admission, and 47 patients were lost to follow-up at one year. Among the patients who were admitted to our hospital after October 2016, two patients declined consent, and one patient presented with severe dementia, precluding evaluation of the gustatory threshold for salty taste. These patients were excluded, and a final total of 277 patients were included in this study (Group A, *n* = 141 and Group B, *n* = 136) (Figure 1).

The patients’ mean age was 69.2 ± 12.2, men comprised 62.1% of patients, the mean BMI was 23.4 ± 4.3 kg/m^2^, and 46.9% of patients had a history of diabetes. The proportions of patients based on CKD stage were G3 (18.0%), G4 (49.1%), and G5 (32.9%). Compared with Group B (historical controls), Group A was significantly younger and had higher BMI and eGFR at admission (Table 1). Given that age and BMI are strongly associated with eGFR, we adjusted for these factors in all analyses performed. The proportions of etiologies of CKD were as follows: diabetic nephropathy, 35.7%; benign nephrosclerosis, 31.0%; chronic glomerulonephritis, 15.5%; and autosomal dominant polycystic kidney disease, 4.3%. No significant differences between groups were observed (Table 1). Compared to Group B, Group A had higher administration rates of calcium channel blockers and glucagon-like peptide-1 (GLP-1) agonists, and lower administration rates of phosphate binder, ion-exchange resin, and carbonaceous oral adsorbents (Table S1).

The gustatory threshold for salty taste at three months after admission was improved relative to that at admission. Salt intake and blood pressure were significantly lower at three months after admission relative to that at admission (Table 2). The median (interquartile range) of urinary sodium (mEq/L)/urinary creatinine (mg/dL) was 1.01 (0.70–1.49) on admission and 0.97 (0.51–1.40) three months after admission.

**Figure 2:**
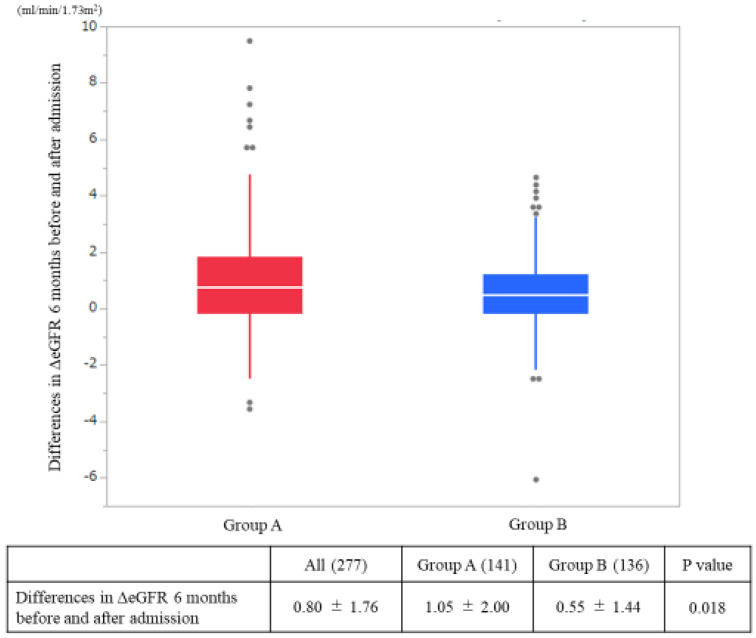
Differences in the estimated glomerular filtration rates (ΔeGFR) on admission and at six months after admission. The *p*-values were statistically analyzed by a Student’s *t*-test.

**Figure 3:**
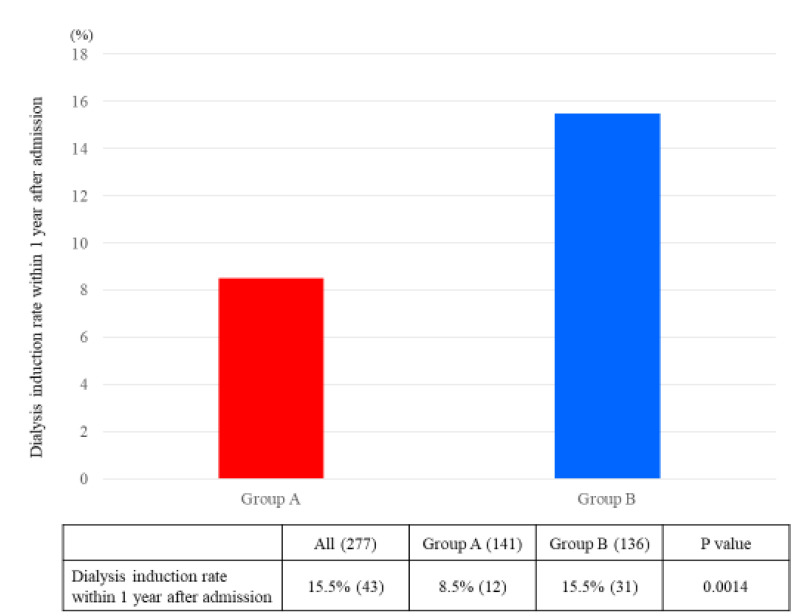
Dialysis induction rate within one year after admission. The *p*-values were statistically analyzed by chi-square test.

The ΔeGFR from admission to six months after admission was significantly greater in Group A than in Group B (A: 1.05 mL/min/1.73 m^2^/month, B: 0.55 mL/min/1.73 m^2^/month; *p* = 0.02) (Figure 2). The hemodialysis induction rate within one year after admission was superior in Group A than in Group B (A: 8.5%, B: 15.5%; *p* = 0.001) (Figure 3).

Propensity score matching was performed, and 105 patients were extracted from each group. Score-matched patients’ backgrounds are shown in the Supplemental Material (Table S2). In addition to the eGFR, there was no significant difference between Group A and Group B in terms of CKD stages (Group A; G3: 16.2%, G4: 51.4%, and G5: 32.4%, Group B; G3: 14.3%, G4: 52.4%, G5: 33.3%). Despite performing propensity score matching, the ΔeGFR results and hemodialysis induction rate exhibited similar tendencies (Table 3).

Univariate and multivariate logistic regression analyses revealed that the rate of hemodialysis initiation within one year was significantly lower in Group A than in Group B (Table 4). Gustatory threshold testing for salty taste was only conducted in Group A, revealing that the mean threshold for salty taste exceeded the normal range.

Stratified analysis by CKD stage revealed that relative to Group B, Group A was superior based on the ΔeGFR in patients with CKD stage 4 and dialysis initiation rates within one year after admission in patients with CKD stage 5 (Table S3). During the observational period, we did not detect any occurrence of severe dehydration or hyperkalemia.

## 4. Discussion

Our findings suggested that educational hospitalization with enhanced salt reduction guidance may reduce the risk of ESRD. Compared with Group B, Group A exhibited a significant improvement in eGFR and a lower dialysis induction rate. Propensity score-matched comparisons and multivariate logistic regression analysis revealed similar results. In addition, the gustatory threshold for salty taste in Group A improved, and the amount of salt intake and blood pressure decreased at three months after admission. Significant differences in the ΔeGFR in patients with CKD stage 4 and dialysis induction rates in patients with CKD stage 5 were observed between groups. We, therefore, speculate that evaluation of the gustatory threshold for salty taste may be effective even in advanced CKD patients.

Medical professionals, including doctors, nurses, pharmacists, and dietitians, engage in patient education programs. Multidisciplinary cooperation in educating patients with CKD is recommended to attenuate the progression of renal dysfunction [15]. The results of this study suggest that the evaluation of gustatory threshold for salty taste to promote salt restriction may alleviate the progression of renal dysfunction. The benefits of salt restriction in patients with CKD are controversial. Some reports have supported salt restriction in patients with CKD. For example, according to a prospective cohort study that was conducted with 3757 patients, high sodium excretion was associated with the risk of cardiovascular disease [16]. Another report showed that salt restriction in patients with CKD had a positive impact on blood pressure, eGFR, *N*-terminal pro-brain natriuretic peptide, and urinary protein ratio [17].

Other reports have not indicated a significant association between urinary sodium and CKD progression or prognosis [18,19]. In addition, excessive salt restriction to less than 3 g per day increased the composite outcome of death and major cardiovascular events [20]. Another study proved that salt restriction of 2.08 g per day was a risk for CKD progression [21].

Consequently, the association between salt restriction and the progression of renal dysfunction remains to be elucidated.

The significant improvement in the ΔeGFR and significantly lower dialysis induction rate in Group A may be considered a result of multi-disciplinary educational intervention, including salt restriction, which induced renal protection by lowering intraglomerular pressure, enhancing the effect of RAS inhibitors and diuretics, and preventing volume overload. Moreover, several factors had recently changed, including patient education of CKD, skills and awareness of medical staff about CKD, and administered drugs; these factors may have also affected the outcome. Notably, there were tendencies for patients in the two groups to attenuate their eGFR after the education programs. During the program, we often arranged prescriptions. For example, decreasing renin-angiotensin system inhibitors if patients had lower blood pressure or had no proteinuria. We also recommended that they did not take nonsteroidal anti-inflammatory drugs if the drugs were not mandatory. In addition, there was the possibility that patients who suffered from acute kidney injury to some extent were included in the study. Moreover, we recommended that patients drunk a suitable amount of water to avoid dehydration unless they had to restrict water. Following this, the eGFR after the education program might have alleviated in both groups.

Patients with CKD have fewer opportunities to receive education programs, especially for diet [5]. Effective diet guidance in clinical practice is challenging. The effects of diet guidance will differ depending on the content of the education programs, and requires time and skills. Furthermore, the effects may differ depending on patients’ attitudes, medical knowledge, and current medical condition. Although the type of diet guidance that would be most effective at halting disease progression remains unknown, this study demonstrated that salt restriction based on gustatory threshold for salty taste evaluation had a positive impact on CKD progression. The patients who received education on CKD acquired knowledge on renal diseases and their clinical conditions during their hospital stay and were made aware of their condition based on the diet guidance with gustatory threshold for salty taste, which may have altered their behaviors in daily life. Patients with CKD are known to have taste disturbances, and education around salt restriction may change their threshold for salty taste [22]. In this study, the improved threshold for salty taste may have affected the dialysis induction rate, ΔeGFR, and the amount of salt consumption.

It is well established that patients with CKD have taste disturbances for salt [23] concomitant with metabolic disturbances, mineral deficiencies including zinc deficiency, peripheral nerve disturbances, adverse drug effects, and uremia [7]. Epithelial Na^+^ channel (ENaC) is expressed in taste cells in the tongue and collecting ducts in the kidney. ENaC enables the perception of salt taste through Nav channel and CALHM1/3 channel signal transduction [24]. Furthermore, angiotensin II acts on AT1 receptors on taste cells and causes disturbances in salt sensitivity. Other mechanisms in the renin-angiotensin-aldosterone system (RAAS) play an important role in salt taste [25]. Salt restriction can improve taste disturbances through the sensitivity of these ion channels and RASS; however, the precise mechanisms remain to be elucidated.

This study had several limitations. First, this study was a non-interventional retrospective analysis conducted in a single center. Second, there were differences in the observational period for Groups A and B. Although CKD treatment policies in Japan did not change dramatically in this timeframe, slight differences in treatment may have affected the outcomes. Even though we conducted a multivariate logistic regression analysis and propensity score matching, there should have been differences which could not be corrected between Group A and Group B. Third, approximately 80% of the patients included in this study had CKD stage 4 or more, and the results obtained in this study cannot be generalized to all patients with CKD. Fourth, although a significant difference in renal outcome was observed between the two groups in this study, the clinical importance of this difference was weak. Since educational programs should be performed with multifactorial aspects, an accumulation of positive effects from several different origins need to be included in the patient education for CKD. The recommendation of salt restriction in this educational program played an important role in lowering blood pressure, but other factors may be associated with this improvement. Hence, it was difficult to prove the direct association between salt restriction and renal outcomes. Fifth, we requested patients be admitted to our hospital for the purpose of education, but there was a possibility that some included patients might have been suffering from acute kidney injury or contemporary renal function decline. Furthermore, the estimated amount of salt intake was evaluated based upon spot urine and might not have reflected the true amount of salt intake.

Despite the limitations of this study, there are several strengths. To our knowledge, there has yet to be a study examining the association between intensive salt restriction based on gustatory threshold for salty taste and renal outcomes. Given that this study was conducted at a single center, the patients’ backgrounds throughout the observational period did not change drastically. Although there were significant differences in the characteristics between the two groups, such as BMI, propensity score matching and multivariate analyses showed the effectiveness of dietary guidance using the gustatory threshold test for salty taste. Furthermore, the education programs were similar, and the method of education did not change, although there was a substantial change in October 2016 in terms of evaluating gustatory threshold for salty taste. In consideration of ethical aspects, we are going to offer Group B patients who are not on maintenance hemodialysis the opportunity to participate in the recent educational program at our hospital.

## 5. Conclusions

Educational hospitalization with enhanced salt reduction guidance may reduce the risk of ESRD, even in patients with advanced CKD. However, the effects of inpatient guidance and salt restriction on renal outcomes in patients with CKD should be investigated more thoroughly in the future.

## Figures and Tables

**Table 1:** Characteristics of patients in the two groups.

	All (277)	Group A (141)	Group B (136)	*p*-Value
Age, years	69.2 ± 12.2	67.7 ± 13.2	70.7 ± 10.8	0.045
Male, %	62.1% (172)	64.5% (91)	59.6% (81)	0.46
Height, cm	160.0 ± 9.3	161.3 ± 61.3	158.6 ± 58.6	0.02
Weight, kg	60.2 ± 13.4	62.9 ± 14.3	57.5 ± 11.8	<0.001
BMI, kg/m^2^	23.4 ± 4.2	24.1 ± 4.8	22.7 ± 3.5	0.007
CKD stage, %	G3: 18.0% (50) G4: 49.1% (136) G5: 32.9% (91)	G3: 24.1% (34) G4: 49.7% (70) G5: 26.2% (37)	G3: 11.8% (16) G4: 48.5% (66) G5: 39.7% (54)	
Primary disease, %	Diabetic nephropathy: 35.7% (99) Nephrosclerosis: 31.0% (86) Glomerulonephritis: 15.5% (43) ADPKD: 4.3% (12) One kidney: 4.3% (12) Others: 9.0% (25)	Diabetic nephropathy: 39.7% (56) Nephrosclerosis: 27.0% (38) Glomerulonephritis: 17.0% (24) ADPKD: 4.3% (6) One kidney: 5.7% (8) Others: 6.4% (9)	Diabetic nephropathy: 31.6% (43) Nephrosclerosis: 35.3% (48) Glomerulonephritis: 14.0% (19) ADPKD: 4.4% (6) One kidney: 2.9% (4) Others: 12.5% (16)	
Diabetes mellitus, %	46.9% (130)	51.1% (72)	42.7% (58)	0.23
BUN, mg/dL	39.7 ± 15.7	39.1 ± 15.8	40.3 ± 15.7	0.55
Cr, mg/dL	2.6 ± 1.1	2.6 ± 1.1	2.5 ± 1.1	0.51
eGFR, mL/min/1.73 m^2^	20.8 ± 10.1	22.4 ± 10.3	19.1 ± 9.6	0.005
Na, mmol/L	139.8 ± 2.5	139.6 ± 2.3	140.2 ± 2.6	0.05
K, mmol/L	4.5 ± 0.5	4.4 ± 0.5	4.6 ± 0.5	0.06
Ca, mg/dL	9.1 ± 0.6	9.1 ± 0.6	9.0 ± 0.6	0.10
P, mg/dL	3.7 ± 0.8	3.7 ± 0.9	3.7 ± 0.8	0.99
UA, mg/dL	7.1 ± 1.6	7.1 ± 1.6	7.1 ± 1.7	0.87
Intact-PTH ^a^, pg/mL	111 (67–177)	104 (63.9–151)	131 (71.7–217)	0.004
HCO_3_^-^, mmol/L	23.1 ± 3.3	23.1 ± 3.4	23.1 ± 3.0	0.98
Alb, g/dL	3.8 ± 0.5	3.8 ± 0.5	3.7 ± 0.5	0.51
LDL-C, mg/dL	96.6 ± 30.1	96.8 ± 28.4	96.4 ± 32.3	0.91
HbA1c, %	6.1 ± 1.0	6.2 ± 1.1	6.1 ± 1.0	0.86
Ferritin ^a^, ng/mL	133 (68–248)	148 (82–273)	111 (49–196)	0.14
Hb, g/dL	11.4 ± 1.7	11.7 ± 1.8	11.1 ± 1.6	0.004
UPCR ^a^, g/gCr	1.5 (0.4–3.3)	1.5 (0.5–3.0)	1.5 (0.3–3.5)	0.58
SBP, mmHg	138 ± 21	138 ± 22	137 ± 20	0.72
DBP, mmHg	76 ± 13	77 ± 12	74 ± 13	0.06

Continuous variables are presented as mean ± standard deviation. Categorical variables are presented as percentages or numbers. The *p*-values were statistically analyzed by a Student’s *t*-test and a Mann–Whitney U-test for the continuous variables and a chi-square test for categorical variables. BMI: body mass index, BUN: blood urea nitrogen, Cr: creatinine, eGFR: estimated glomerular filtration rate, UA: uric acid, PTH: parathyroid hormone, Alb: albumin, LDL-C: low-density lipoprotein cholesterol, HbA1c: hemoglobin A1c, Hb: hemoglobin, UPCR: urinary protein-creatinine ratio, SBP: systolic blood pressure, DBP: diastolic blood pressure, ^a^ median is the interquartile range.

**Table 2:** Gustatory threshold for salty taste, salt intake, and blood pressure at admission and three months after admission in Group A.

	On Admission	3 Months after Admission	*p*-Value
Gustatory threshold for salty taste (mg/cm^2^)	1.02 ± 0.35	0.85 ± 0.28	<0.001
Salt intake (g/day)	8.5 ± 2.0	8.1 ± 2.5	0.04
SBP, mmHg	137 ± 21	133 ± 16	0.003
DBP, mmHg	76 ± 13	74 ± 11	0.02

Continuous variables are presented as mean ± standard deviation. SBP: systolic blood pressure, DBP: diastolic blood pressure. The *p*-values were statistically analyzed by a paired *t*-test.

**Table 3:** Comparison between groups after propensity score matching.

	Group A (105)	Group B (105)	HR	*p*-Value
Dialysis initiation rate, %	9.5% (10)	21.0% (22)	0.40 (0.18–0.89)	0.03
ΔeGFR at admission and 6 months after admission (mL/min/1.73 m^2^/month)	0.94 ± 1.79	0.49 ± 1.50		0.048

The *p*-values were statistically analyzed using a Student’s *t*-test for the continuous variables and a chi-square test for categorical variables.

**Table 4:** Univariate and multivariate logistic regression analyses of the dialysis induction rate.

	Univariate			Multivariate (Model 1)			Multivariate (Model 2)		
	HR	95% CI	*p*-Value	HR	95% CI	*p*-Value	HR	95% CI	*p*-Value
Age, year	0.99	0.96–1.01	0.33	0.98	0.95–1.01	0.13	0.96	0.93–0.99	0.01
Male, %	0.65	0.34–1.26	0.21	0.66	0.34–1.30	0.24	1.15	0.53–2.48	0.72
Enhanced salt reduction guidance	0.32	0.15–0.64	0.001	0.30	0.14–0.61	<0.001	0.35	0.16–0.81	0.01
BMI, kg/m^2^	0.91	0.83–1.00	0.03				0.94	0.85–1.04	0.23
eGFR, mL/min/1.73 m^2^	0.82	0.77–0.88	<0.001				0.81	0.75–0.88	<0.001
Diabetes mellitus, %	0.98	0.51–1.88	0.95						
K, mmol/L	1.37	0.81–2.33	0.24						
Ca, mg/dL	0.38	0.21–0.69	0.002						
P, mg/dL	1.91	1.28–2.86	0.001						
UA, mg/dL	1.03	0.83–1.26	0.80						
Alb, g/dL	0.31	0.17–0.59	<0.001						
LDL-C, mg/dL	0.99	0.98–1.00	0.18						
HbA1c, %	0.87	0.58–1.30	0.48						
Hb, g/dL	0.61	0.49–0.76	<0.001						
UPCR, g/gCr	1.37	1.21–1.55	<0.001						
SBP, mmHg	1.03	1.01–1.05	<0.001						
DBP, mmHg	1.04	1.01–1.07	0.002						

BMI: body mass index, eGFR: estimated glomerular filtration rate, UA: uric acid, Alb: albumin, LDL-C: low-density lipoprotein cholesterol, HbA1c: hemoglobin A1c, Hb: hemoglobin, UPCR: urinary protein-creatinine ratio, SBP: systolic blood pressure, DBP: diastolic blood pressure.

## Supplementary Materials

The following are available online at https://www.mdpi.com/2072-6643/12/9/2703/s1, Table S1: Administration of drugs in the two groups. Table S2: Characteristics of the propensity score-matched cohort; Table S3: Stratified analysis according to the CKD stage.
