# Supplementary material for: Risk Reduction for End-Stage Renal Disease by Dietary Guidance Using the Gustatory Threshold Test for Salty Taste
Source: Nutrients. 2020 Sep 4;12(9):2703. doi: 10.3390/nu12092703 (PMC7551153; doi:10.3390/nu12092703)
Supplement: Supplementary file 1 [file nutrients-12-02703-s001.pdf]

< Supplementary Materials >

Table S1. Administration of drugs in the two groups

	All (277)	Group A (141)	Group B (136)	P value
ACE-I・ARB, %	69.0% (191)	71.1% (101)	66.7% (90)	0.44
CCB, %	71.5% (198)	78.2% (111)	64.4% (87)	0.02
β blocker, %	22.4% (62)	21.1% (30)	23.7% (32)	0.67
Diuretic, %	46.9% (130)	40.9% (58)	53.3% (72)	0.04
Vitamin D, %	24.9% (69)	22.5% (32)	27.4% (37)	0.40
Phosphate binder, %	9.7% (27)	3.5% (5)	16.3% (22)	<0.001
Potassium binder, %, %	22.4% (62)	16.9% (24)	28.2% (38)	0.03
Bicarbonate, %	22.7% (63)	20.4% (29)	25.2% (34)	0.39
ESA, %	26.0% (72)	20.4% (29)	31.9% (43)	0.04
Carbon adsorbent, %	21.7% (60)	14.8% (21)	28.9% (39)	0.005
Insulin, %	14.8% (41)	15.5% (22)	14.1% (19)	0.87
GLP-1 agonist, %	5.1% (14)	8.5% (12)	1.48% (2)	0.01
Oral antidiabetic drug, %	27.1% (75)	31.7% (45)	22.2% (30)	0.08
Statin, %	45.1% (125)	47.2% (67)	43.0% (58)	0.55

Categorical variables are presented as percentage or number. P-values were statistically analyzed using the chi-square test.

ACE-I: Angiotensin-converting enzyme inhibitor, ARB: Angiotensin receptor blocker, ESA: erythropoiesis stimulating agent, GLP-1: Glucagon-like peptide-1

Table S2: Characteristics of the propensity score-matched cohort

	Group A (105)	Group B (105)	P value
Age, year	69.8 ± 12.2	69.6 ± 11.2	0.92
Male, %	61.0% (64)	63.8% (67)	0.78
BMI, kg/m^2	23.4 ± 4.7	23.2 ± 3.5	0.85
Diabetes mellitus, %	43.7% (49)	46.7% (49)	1.0
eGFR, ml/min/1.73 m^2	20.4 ± 9,2	20.0 ± 9.3	0.74
CKD stage, %	G3: 16.2%<br>G4: 51.4%<br>G5: 32.4%	G3: 14.3%<br>G4: 52.4%<br>G5: 33.3%	
Hb, g/dL	11.3 ±1.3	11.3 ± 1.6	0.81
SBP, mmHg	137.3 ± 21.6	138.1 ± 21.7	0.76
UPCR[a], g/gCr	1.46 (0.60 - 3.15)	1.49 (0.25 - 3.84)	0.70

Continuous variables are presented as mean standard deviation and categorical variables as percentage or number. P-values were statistically analyzed using the Student's t-test and Mann-Whitney U test for continuous variables and chi-square test for categorical variables.

BMI: body mass index, eGFR: estimated glomerular filtration rate, Hb: hemoglobin, SBP: systolic blood pressure, UPCR: urinary protein-creatinine ratio

[a] Median (interquartile range)

Table S3: Stratified analysis according to the CKD stage

a) Changes in ΔeGFR at admission and 6 months after admission.

CKD stage	Group A (141)	Group B (136)	P value
G3	0.81 ± 2.49	-0.29 ± 1.95	0.12
G4	1.15 ± 2.02	0.44 ± 1.42	0.02
G5	0.16 ± 1.10	-0.01 ± 0.71	0.38

P-values were statistically analyzed using the Student's t-test.

b) Dialysis induction rate within one year after admission

CKD stage	Group A (141)	Group B (136)	P value
G3	2.9% (1)	6.3% (1)	0.54
G4	4.3% (3)	7.6% (5)	0.48
G5	21.6% (8)	46.3% (25)	0.03

P-values were statistically analyzed using the chi-square test.

eGFR: estimated glomerular filtration rate; CKD: chronic kidney disease
